# Subthalamic nucleus synchronization between beta band local field potential and single‐unit activity in Parkinson's disease

**DOI:** 10.14814/phy2.16001

**Published:** 2024-05-02

**Authors:** Eric Bayman, Keanu Chee, Madelyn Mendlen, Daniel J. Denman, Rex N. Tien, Steven Ojemann, Daniel R. Kramer, John A. Thompson

**Affiliations:** ^1^ Department of Neurosurgery University of Colorado Anschutz Medical Campus Aurora Colorado USA; ^2^ Department of Neurophysiology and Biophysics University of Colorado Anschutz Medical Campus Aurora Colorado USA; ^3^ Department of Neurology University of Colorado Anschutz Medical Campus Aurora Colorado USA

## Abstract

Local field potential (LFP) oscillations in the beta band (13–30 Hz) in the subthalamic nucleus (STN) of Parkinson's disease patients have been implicated in disease severity and treatment response. The relationship between single‐neuron activity in the STN and regional beta power changes remains unclear. We used spike‐triggered average (STA) to assess beta synchronization in STN. Beta power and STA magnitude at the beta frequency range were compared in three conditions: STN versus other subcortical structures, dorsal versus ventral STN, and high versus low beta power STN recordings. Magnitude of STA‐LFP was greater within the STN compared to extra‐STN structures along the trajectory path, despite no difference in percentage of the total power. Within the STN, there was a higher percent beta power in dorsal compared to ventral STN but no difference in STA‐LFP magnitude. Further refining the comparison to high versus low beta peak power recordings inside the STN to evaluate if single‐unit activity synchronized more strongly with beta band activity in areas of high beta power resulted in a significantly higher STA magnitude for areas of high beta power. Overall, these results suggest that STN single units strongly synchronize to beta activity, particularly units in areas of high beta power.

## 1 INTRODUCTION

Parkinson's disease (PD), a neurodegenerative disease which manifests as tremor, rigidity, bradykinesia and other motor and non‐motor symptoms (Krack et al., 1998; Limousin et al., 1995, 1998), is marked by a loss of midbrain dopaminergic neurons and their projections into the basal ganglia. This loss leads to downstream changes in connected areas that can be targeted for treatment of PD, such as the subthalamic nucleus (STN). In the STN of PD patients, abnormalities have been characterized in both single‐unit neuronal spiking activity and local field potential (LFP) oscillations. Despite these findings, it has been challenging to causally link firing rate changes, bursting, or synchronization to PD motor symptoms, suggesting that a combination of these variables may explain some of these clinical paradoxes (Bevan et al., 2002; McGregor & Nelson, 2019).

In addition to the characterization of single‐unit activity in PD STN specifically associated with surgical mapping, exploration of STN‐LFP has led to additional pathophysiological insights. Most notably, LFP measurements demonstrate increased power in the beta frequency band (12–30 Hz) in PD patients (Alonso‐Frech et al., 2006; Brown & Williams, 2005; Fogelson et al., 2006; Kühn et al., 2004). These oscillations are suppressed with dopaminergic medications (Brown et al., 2001; Priori et al., 2004) and DBS (Bronte‐Stewart et al., 2009; Eusebio et al., 2011; Giannicola et al., 2010; Quinn et al., 2015; Whitmer et al., 2012; Wingeier et al., 2006), and the degree of suppression of beta activity has been correlated to the degree of clinical improvement in motor symptoms (Kühn et al., 2008, 2006; Little et al., 2013; Ray et al., 2008). There is a growing body of literature identifying abnormalities in the beta frequency range in the STN of PD patients, but the relationship between beta power, spiking, and PD motor symptoms is still not clearly understood.

Although LFP is thought to result from aggregate electric currents and subthreshold fluctuations, individual spikes themselves are not thought to contribute significantly to LFP because of the short time course of neuron discharge and limited synchronized firing under physiological conditions (Buzsáki et al., 2012). Bursting STN neurons, as well as beta oscillations, have been localized to the dorsolateral STN, where beta power has been shown to be the highest (Kühn et al., 2005; Seifried et al., 2012; Trottenberg et al., 2007; Wingeier et al., 2006). These findings demonstrate the difficulty in identifying a causative relationship between beta power, single‐neuron activity, and PD motor symptoms.

One electrophysiological method for inferring the relationship between neuronal spiking and LFP is the use of spike‐triggered average LFP (STA‐LFP). STA‐LFP is the average of the LFP taken at the times of spike occurrences (Jin et al., 2008; Nauhaus et al., 2009). Features of the LFP that happen at a consistent time around each spike will be preserved in the spike‐triggered average whereas unsynchronized LFP elements will not. This method of analysis has been used to measure postsynaptic LFP changes resulting from single‐neuron activity, providing a temporal relationship between spiking activity and LFP signatures (Lee et al., 2023). The objective of this study is to apply STA‐LFP analysis using spikes and LFP recorded in the STN of PD patients during rest to evaluate the synchronization between the two signals. This can be used as a measure of connection between STN neurons and LFP of regions within the STN. Comparing the STA‐LFP and the beta power between different conditions can reveal information about spike synchronization to beta oscillations.

We compared STA‐LFP magnitude and percent beta power during awake DBS surgery in PD patients in the following conditions: STN versus other structures along the recording path, dorsal versus ventral STN, and areas of high versus low beta power recordings inside the STN. We expected that an increase in beta power in the STN would correspond with a higher magnitude STA‐LFP suggesting single‐unit activity is synchronized to the increased beta oscillations seen in PD.

## 2 METHODS

### 2.1 Study participants and surgical methods

Surgical methods were standard for deep brain stimulation surgery and have been described elsewhere (Benabid et al., 2009), and all surgeries were performed at the University of Colorado Anschutz Medical campus between 2017 and 2019. This study was performed in accordance with the Colorado Multiple Institutional Review Board (COMIRB #16‐1060) and written consent was obtained from all subjects preoperatively. Briefly, surgical targeting was performed on preoperative imaging and based on indirect targeting methods and refined based on direct visualization using magnetic resonance imaging (MRI). Surgery was performed awake with a stereotactic frame. One to three recording electrodes (determined by the operating surgeon) were advanced from 25 mm above the surgical target through the STN (Abosch et al., 2002, 2013; Kramer et al., 2010). No analysis was performed using the macroelectrode (3 mm from the tip) because the final recording was generally made 0.5–2 mm below the inferior border of the STN. Therefore, the macroelectrode would fail to record within the inferior portion of STN and would miss the substantia nigra pars reticulata (SNr) completely. There were also noted to be fewer noise artifacts in the microelectrode recordings. The surgical target was initially located using preoperative MRI and STN entry/exit points were determined by clinicians based on microelectrode recordings (Abosch et al., 2013). Operative notes were used to determine whether recordings occurred within STN and whether they occurred in the dorsal or ventral region based on distance from target, adjusted for the microelectrode recording guided entrance and exit of the STN.

### 2.2 Electrophysiology data processing

Raw and spiking signals were sampled at 44 kHz by a 16‐bit A/D converter (using ±1.25 V input range, that is, ∼2 μV amplitude resolution) and band‐passed from 0.7 to 9000 Hz using a hardware four‐pole Butterworth filter (AlphaOmega, NeuroOmega, Nazareth, Israel). Electrophysiological microelectrode recordings (MER) began at 25 mm above the ventral border of the STN and advanced in steps of 100–1000 μm. The dorsal and ventral borders of the STN, as well as entry into the SNr, were assessed by a neurosurgeon, neurologist, and neurophysiologist based on auditory and visual analysis of single‐neuron activity with coincident kinematic motor testing. All subsequent analyses were computed offline. LFP recordings were inspected and discarded if significant artifacts were present. Artifacts primarily consisted of impedance testing or other noise which can be identified by signal that is orders of magnitude larger than uncontaminated LFP recordings (see Appendix S1 for an example). Visual inspection showed that uncontaminated LFPs had values below 300 μV so recordings with values above 300 μV were discarded (*n* = 100/1692 recordings). 60 Hz line noise and harmonics were removed from the remaining LFP recordings using a notch filter (MATLAB; BANDSTOPIIR function) (Air et al., 2012). Spike clustering analysis was performed on the raw voltage data collected from NeuroOmega (Alpha Omega, Alpharetta, GA) using open‐source MATLAB software Wave_Clus, with an unsupervised spike‐sorting method with the following parameters: sampling rate = 44,000 Hz, minimum threshold for detection = −3 × 𝑠𝑡𝑑, maximum threshold = −30 × 𝑠𝑡d, and isolation threshold 3 ms (Quiroga et al., 2004). Clustered waveforms were manually inspected and excluded if a spike amplitudes were close to background noise or if the waveforms appeared non‐physiological (see Appendix S2 for an example, confirmed by two evaluators; *n* = 740/1696 clusters excluded).

### 2.3 STA analysis

To conduct STA analyses, LFP recordings were filtered to the frequency range of interest (e.g., 12–30 Hz). Recordings were first high passed (12 Hz) then low passed (30 Hz) using BANDSTOPIIR, FILTFILT (MATLAB, 2022). Analysis of STA‐LFP for STN versus other subcortical structures was also performed in the gamma (30–100 Hz), alpha (8–12 Hz), theta (4–8 Hz), and delta (1–4 Hz) frequency ranges. Of these frequency ranges, the STA‐LFP extracted in the LFP beta frequency range appeared to have the highest amplitude, which we expected based on previous studies on beta power in PD patients (see Appendix  S3). Signals 0.5 s long were extracted from each LFP recording centered around the corresponding spike times (Kühn et al., 2005). Spikes occurring during the first or last 500 milliseconds of the recording were excluded to reduce the impact of transient events on the LFP tracing (Snyder & Smith, 2015). Linear voltage offset was subtracted to center the LFP segment around 0 (Snyder & Smith, 2015). This process was repeated for every LFP recording in the condition, and the resulting 0.5 s LFP segments at each spike time point were averaged to create the spike‐triggered grand average. To create a non‐parametric shuffle‐derived comparison, the same analysis was performed with an equivalent number of random spike times for each LFP recording, and an STA derived from these randomly generated spikes.

Using a *t*‐test, the magnitude of the STA at each time point was tested for statistical significance versus the magnitude of the STA at the same time point from the corresponding matrix created using random spike times. For each statistically significant time point, the absolute value of the magnitude was recorded. Finally a root‐mean‐square magnitude value was calculated for the STA‐LFP of each recording using each magnitude value from each significant time point. When spikes and LFP are recorded from the same microelectrode, spike components before, during, and after can cause artifacts in LFP recordings. This primarily affects higher frequencies (0.8‐5 kHz) and can be seen down to 100 Hz (Ray, 2015). This analysis was focused on frequency ranges below 100 Hz to avoid contamination. We used an artifact rejection method to remove this contamination and noted that below 100 Hz the artifact rejection did not alter the appearance of the signal (See supplementary figures in the Appendix S4) (Boroujeni et al., 2020).

### 2.4 Bandpower analysis

To assess whether recordings occurred in high or low beta power localized regions of the STN, we used the BANDPASS BANDPOWER function (MATLAB, 2022) to find the percent beta power (Kühn et al., 2005). First, we calculated the band power in each recording from 0 to 100 Hz and considered this the total power. Then for that same recording, we calculated the band power at each integer frequency value between 12–30 Hz ± 0.5 Hz (for example, to find the power at 13 Hz, we found the band power from 12.5 Hz to 13.5 Hz). Finally, we determined the percent beta power at each integer frequency value using the equation:

$$\begin{array}{c} \%\;\text{Power}\;\mathrm{at}\;a\;\mathrm{given}\;\mathrm{frequency}\\ =\frac{\text{Integer frequency bandpower}}{\text{Total bandpower}\;\left(0–100\mathrm{Hz}\right)}\times 100 \end{array}$$

This process was conducted to find the percent beta power at each integer frequency from 12 to 30 Hz in each high and low beta recording. A two‐sided Wilcoxon rank sum test was used on all recordings in each condition to determine the significantly different frequencies between each condition. The following process was used to find the overall percent beta power for each condition; the total percent beta power was calculated for each recording individually; then, each of these values was averaged to determine the mean percent beta power for a condition.

$$\text{Percent beta power}=\frac{\text{Bandpower}\;12–30\mathrm{Hz}}{\text{Bandpower}\;0–100\mathrm{Hz}}\times 100$$

Since beta is a broad band and previous studies have shown a lack of consistency in which frequency in the beta band is most important (van Wijk et al., 2016), we determined the frequency at which the highest peak within the beta band was most active for that patient and that recording. We quantified beta peaks estimating percent beta power at each frequency (Kühn et al., 2005). The highest beta peak was measured for each recording, and each patient's recordings were sorted into quartiles based on the peak of highest percent power. For the combined analysis, one recording was selected from each patient's first and last quartile of recordings to create the high and low beta power conditions. Using one recording from each patient for each condition minimized the possibility that one patient's percent beta power would be significantly higher or lower than the others, and therefore would make up the majority of either the high or low beta power condition.

### 2.5 Statistical analysis

To compare the STA‐LFP magnitude difference between conditions, we first used a One‐sample Kolmogorov–Smirnov test (ktest function, MATLAB, 2022) which determined that the magnitudes were not normally distributed. Therefore, a two‐sided Wilcoxon rank sum test was used to test for significance. To determine significant time points, we compared each magnitude value that was averaged to create a time point of the STA‐LFP to the corresponding magnitude values at the same time point of the shuffle‐derived baseline using a Wilcoxon rank sum test. To compare the difference in the root‐mean‐square magnitude between conditions, we used a Wilcoxon rank sum test on the root‐mean‐square magnitudes from each recording included in the condition.

## 3 RESULTS

We collected intraoperative electrophysiological recordings from 25 PD patients (18M, 7F) refractory to medical treatment undergoing stereotactic DBS implantation surgery. The average age at diagnosis was 57.3 ± 7.9 years with PD duration of 9.7 ± 4.9 years at the time of surgery. Microelectrode recordings were obtained at intervals of 0.1–1 mm with recordings averaging 26.4 s (±1.3 s). Six percent of recordings were discarded due to noise contamination (see Section 2 for explanation). A total of 1634 single units were extracted using Wave_Clus offline sorting with an average of 557 spikes per unit (±1054 SD; minimum = 26; maximum = 1149). We compared the magnitude of spike‐triggered average LFP potential and the difference in beta power in three conditions: STN versus other structures, dorsal versus ventral STN, and high versus low beta power recordings within STN.

### 3.1 STA‐LFP comparing STN with other basal ganglia structures

**FIGURE 1:**
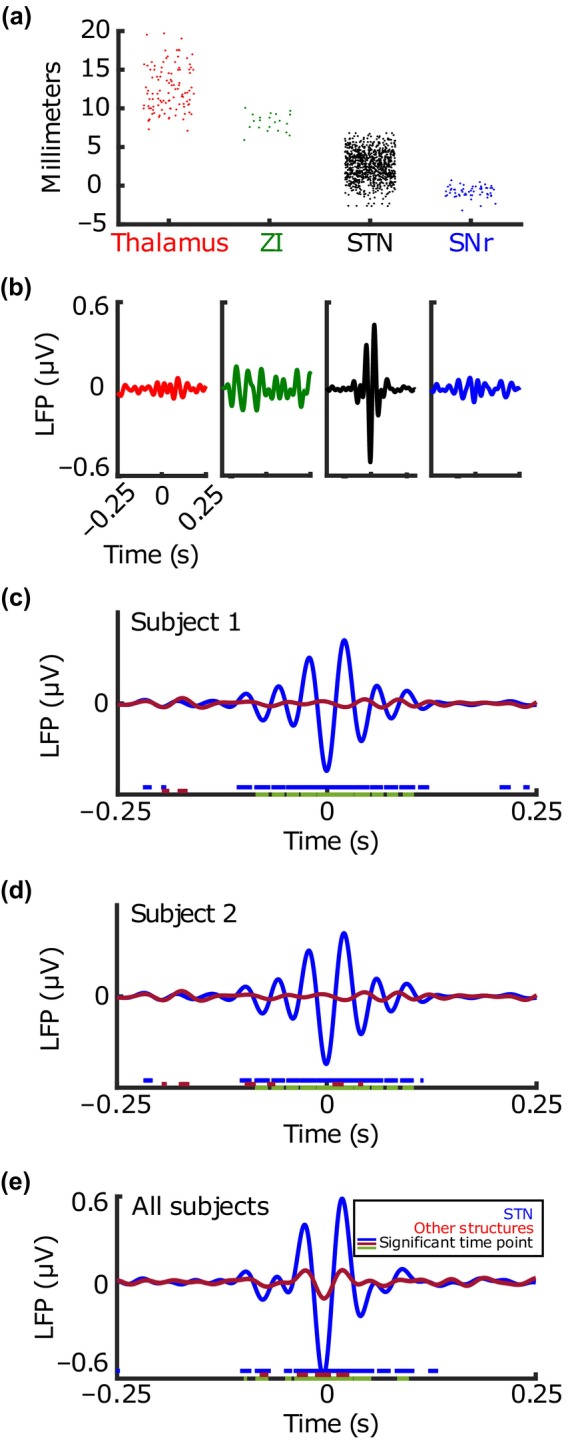
Subthalamic nucleus (STN) compared to other nearby structures. (a) Recording location (represented by a point) in millimeters above preoperative target for Thalamus (*n* = 107), Zona Incerta (ZI, *n* = 21), STN (*n* = 984), and Substantia Nigra pars reticularis (SNr, *n* = 56) recordings. (b) All spike‐triggered average LFP (STA‐LFP) tracings, of all units, averaged together, grouped by structure. Colors correspond to structures in (a). Raw LFP tracings were band passed from 12 to 30 Hz in MATLAB to create 12–30 Hz STA‐LFPs. (c‐d) STA‐LFP tracings for representative subjects. Shading represents ±1 standard error (shading can be difficult to visualize due to low standard error). Legend in (e) corresponds to (c, d). Underlining represents statistically significant time points based on root‐mean‐square analysis of spike‐triggered average (STA) amplitude. Blue and red: Wilcoxon rank sum tests for STN and other structure conditions as compared to an STA constructed from randomly chosen spike time points (not pictured as it was near 0 for all conditions tested). Green: Wilcoxon rank sum test between conditions. (e) Mean STA‐LFP for all recordings inside STN versus other structures with shading and significant time points as in (c‐d). STN recordings were associated with larger magnitude STAs than other structures suggesting individual spikes are more aligned to beta phase inside STN.

Previous studies have shown that there is increased beta power in the STN of PD patients (Kühn et al., 2004) and that single‐unit activity in STN is related to tremor, rigidity, and bradykinesia (Steigerwald et al., 2008). Nine hundred and eighty‐four recordings were taken from the STN, 107 from the thalamus, 21 from zona incerta (ZI), and 56 from SNr (see Figure 1a). There was an average of 671 (±1140) neurons isolated from STN recordings and 383 (±882) isolated from other structures.

There was no significant difference in mean percent beta power between the STN and other structures (10.03% ± 8.32 vs. 9.98% ± 7.30, *p* = 0.30). The magnitude of the spike‐triggered average LFP inside the STN was significantly higher than outside (0.2 μV ± 1.03 vs. 0.04 μV ± 0.71, respectively, *p* = 0.005) (Figure 1e).

### 3.2 Dorsal versus ventral STN

**FIGURE 2:**
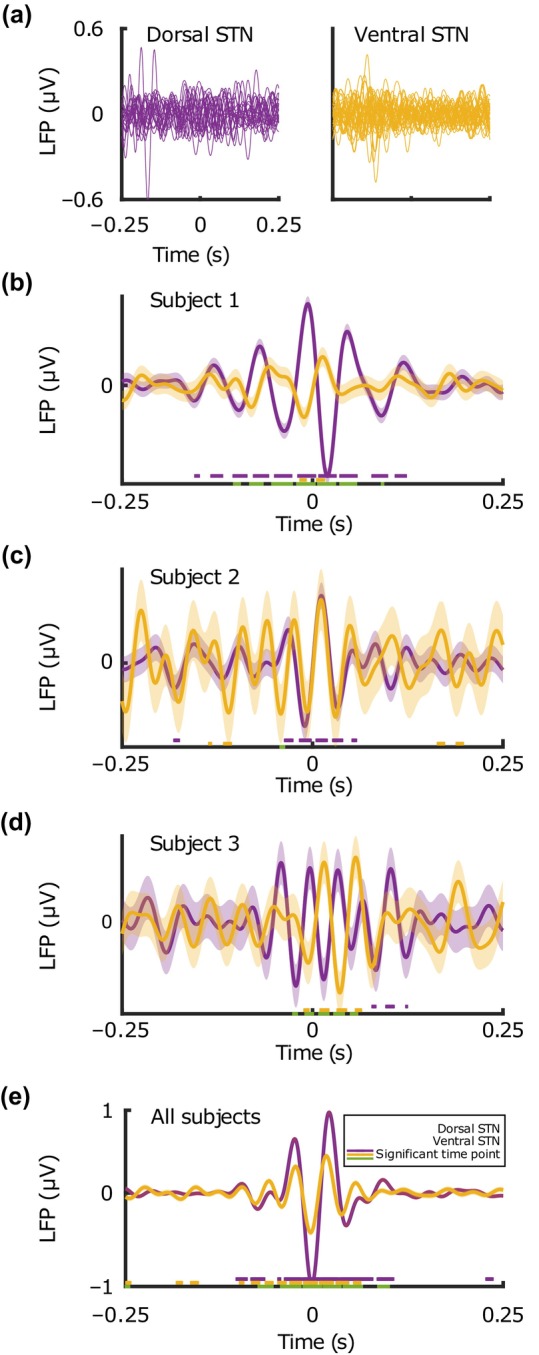
Spike‐triggered averages (STA) of the local field potential (LFP) in the beta band as compared in the dorsal and ventral subthalamic nucleus (STN). (a) All spike‐triggered average LFP (STA‐LFP) tracings created for each recording in the dorsal and ventral conditions. (b‐d) STA‐LFP tracings for representative subjects. Shading represents ±1 standard error. Legend in (e) corresponds to (b,‐d). Underlining as in Figure 1. Purple and yellow: Wilcoxon rank sum tests for dorsal and ventral STN conditions as compared to an STA constructed from randomly chosen spike time points (not pictured as it was near 0 for all conditions tested). Green: Wilcoxon rank sum test between conditions. (e) Mean STA‐LFP for all recordings inside dorsal versus ventral STN with shading and significant time points as in (b‐d). Although dorsal STN recordings had significantly higher percent beta power, the STA magnitude was not significantly different between the two conditions, suggesting that even ventral STN recordings with lower percent beta power are still aligned with beta oscillations in the LFP.

The dorsal and ventral borders of STN were calculated by the total length of the recording, determined by clinical assessment. For simplicity, the dorsoventral axis was bisected into equal compartments. In total, 504 recordings were collected from dorsal STN and 443 from ventral. There was an average of 923 (±1499) neurons isolated from dorsal STN recordings and 446 (±563) isolated from ventral STN recordings. There was a significant difference in percent beta power between the dorsal and ventral STN (10.96% ± 9.51 vs. 9.07% ± 6.92, respectively, *p* = 0.01). However, there was not a significant difference in the mean STA‐LFP magnitude (0.23 μV ± 1.21 vs. 0.11 μV ± 0.78, *p* = 0.12, Figure 2e).

### 3.3 High versus low beta power recordings

**FIGURE 3:**
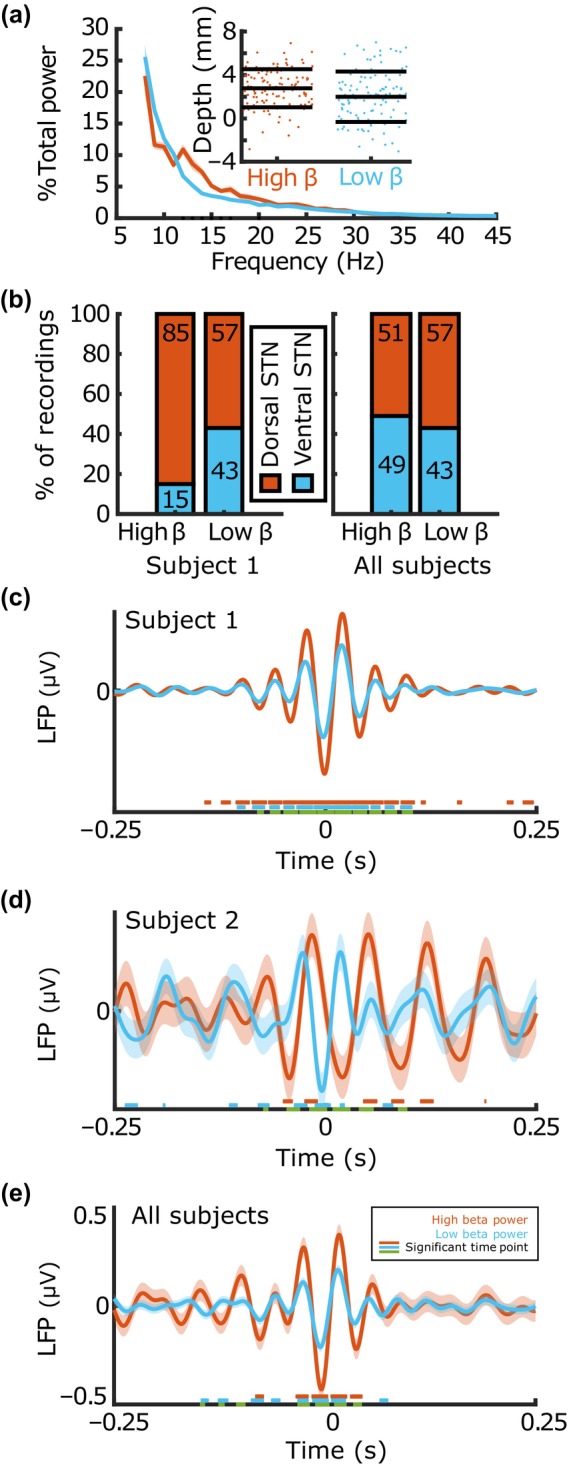
Comparison of beta band spike‐triggered average (STA) in areas of high versus low beta power recordings in the subthalamic nucleus (STN). (a) Mean percent total power across all frequencies, grouped by high (top quartile) and low (bottom quartile) beta power conditions. Orange: High beta. Blue: Low beta. Shading represents ±1 std. error. Inset) Locations for high beta and low beta recordings. Black lines represent mean recording location and ±1 standard error. (b) Left: Representative subject showing percent of high and low beta recordings located in the dorsal and ventral STN. Right: All subjects showing the percentage of high and low beta recordings occurring in the dorsal and ventral STN. Unexpectedly, the highest and lowest beta power recordings were relatively evenly distributed between the dorsal and ventral STN. (c‐d) Spike‐triggered average LFP (STA‐LFP) tracings for representative subjects. Shading represents ±1 std. error. Legend in (e) corresponds to (c‐d). Underlining represents statistically significant time points as in Figure 1. Orange and blue: Wilcoxon rank sum tests for high and low percent beta conditions as compared to an STA constructed from randomly chosen spike time points (not pictured as it was near 0 for all conditions tested). Green: Wilcoxon rank sum test between conditions. (e) Mean STA‐LFP for all recordings inside high versus low beta with shading and significant time points as in (c‐d). High and low percent beta recordings were equally likely to be found in dorsal versus ventral STN. High beta power recordings were associated with larger magnitude STAs suggesting individual spikes are more aligned to beta phase in areas of high beta power.

Although dorsal and ventral STN showed differences in beta power, we also sought to isolate the region of the STN with the highest beta power, independent of location. We found that high beta power recordings (upper quartile of beta power) were equally likely to be found in dorsal and ventral STN (51% vs. 49% of high beta recordings, respectively), and low beta recordings (bottom quartile of beta power) followed a similar trend (57% dorsal vs. 43% ventral, see Figure 3b). When comparing high versus low percent beta power at each frequency from 12 to 30 Hz, the significantly different frequencies were the low beta frequencies, or 12–20 Hz, consistent with previous studies that found that low beta was correlated to motor symptoms in PD (Marceglia et al., 2006; Priori et al., 2004) (Figure 3a). From the 25 high and 25 low beta recordings, we extracted 92 units from the high beta LFPs with an average of 314 spikes each (±397) and 115 units from the low beta LFPs with an average of 909 (±1630) spikes each. STA‐LFP magnitude was significantly higher in the high beta recordings than low beta recordings (0.25 μV ± 1.0318 vs. 0.12 μV ± 0.55, respectively, *p* = 1.1537e^−4^, Figure 3e).

## 4 DISCUSSION

Although both single‐unit activity and LFP in the beta frequency band are related to PD symptoms, the relationship between the two neural signals remains underexplored. Here, we utilized concurrent recordings in the STN of PD patients during DBS surgery to evaluate the spike‐triggered average LFP as a marker of synchronization.

We found no difference in mean beta power between structures inside versus outside STN but did note a significant difference in the magnitude of the STA‐LFP in the beta band. We did not find a difference in the magnitude of the beta band STA‐LFP in the dorsal and ventral STN but did find a difference for areas of high beta power compared to low beta power. Together, we find an overall dissociation between beta power and the beta band STA‐LFP, but a high association between these two measures for STN regions with high beta power, suggesting the single‐unit activity and beta power in these areas is highly synchronized.

Previous studies have shown that beta power is increased inside the STN of PD patients (Brown, 2003; Dostrovsky & Bergman, 2004; Kühn et al., 2005), which was not consistent with our recordings. In the STN, as compared to the extra‐subthalamic locations, the beta power was not significantly different, although this may be due to averaging the beta power over the entire STN. However, the STA‐LFP magnitude was significantly different at 12–30 Hz. These findings suggest that STN neurons may fire more synchronously with beta oscillation phase than neurons in other structures do.

Beta band activity has been shown to synchronize with high‐gamma oscillations in the motor cortex (Shimamoto et al., 2013), but not necessarily to synchronize internally with the STN neurons themselves. Weinberger et al. (2006) showed that 28% of the neurons found within STN displayed significant oscillations in the beta frequencies, the majority of which were found in dorsal STN. Our results corroborate previously published work with beta oscillations correlated between spike and LFP activity as we see that on average, the STN neurons are synchronized to beta activity. Since the STA‐LFP takes all neurons within the STN, with a diverse set of firing rate patterns, waveforms, and activity, this suggests that the STN shows a high degree of synchronization to beta band activity. Although this could be from a small area of strongly synchronized activity, when we examined both dorsal and ventral STN, and high and low beta power areas of the STN, all showed a high STA‐LFP magnitude in the beta band, suggesting the entire STN shows a phase‐relationship between single‐unit and beta activity in PD patients.

By averaging STA‐LFPs across structures, we could lose synchronous but phase‐independent STAs in other structures. There is no way to conclude from this analysis that the other areas are not beta‐synchronous, just that they are not beta phase locked the way STN is across the neural population. Based on the oscillatory STA‐LFP tracings, we can infer that STN neurons are closely linked to the 12–30 Hz frequency band. Since this band is associated with PD symptoms and thought to prevent the brain from initiating a different sensorimotor state (Engel & Fries, 2010), it may further implicate these individual neurons in this process.

We found that although dorsal STN had significantly higher beta power, the difference in magnitude between the STA‐LFP of the two conditions was not significantly different. This suggests that although dorsal STN has higher average beta frequency power, as expected based on previous studies (Kühn et al., 2005; Weinberger et al., 2006), neurons found within both dorsal and ventral STN are closely synchronized to beta oscillations in PD. This suggests that the relationship between beta activity and spiking is consistent throughout the STN.

Regions of high versus low beta power were expected to be located in the dorsal versus ventral STN, respectively (Kühn et al., 2005; Weinberger et al., 2006). However, we found that high and low beta power recordings were distributed equally throughout dorsal and ventral STN. Traditionally, the dorsal STN has been associated with improved motor symptoms when targeted with DBS (Hamel et al., 2004), and since beta power has also been associated with improved motor symptoms (Brown et al., 2001), the two have been assumed to be linked. Here, we saw a dissociation between the anatomic region and beta power activity. In the STA‐LFP magnitude analysis, high beta power recordings produced a significantly higher magnitude STA‐LFP than low beta power recordings, suggesting synchronization to beta oscillation phase is associated with beta power. Low beta power recordings appear to be phase locked as well to a lesser extent. Since beta power was not strictly associated with STA‐LFP in our other analyses, this suggests that in the STN, the single‐unit activity is tightly linked to beta power but not anatomic subregion. Presumably, a targeting of beta peaks within STN is therefore a targeting of beta‐synchronized spiking activity.

We also noted that the difference in magnitude of the STA‐LFP tracings in the “high versus low beta power” condition was less than the difference in magnitude of the STA‐LFP tracings in the “STN versus other structures” condition despite a much more significant difference in beta power. This is evidence that STN neurons are much more closely coupled to beta oscillation phase compared to select other structures, with areas of high and low beta throughout dorsal and ventral STN. Due to its correlative nature, it is still unclear whether STA‐LFP signatures are a result of single‐unit activity or incidental correlations (Teleńczuk et al., 2017).

The spike‐triggered LFP is a measure of the linear correlation between continuous LFP signal and single‐unit activity. Here, we showed that whether the magnitude of the beta band STA‐LFP is significantly different between two conditions can be independent of whether the percent beta power is significantly different between those conditions. We found that in certain conditions, although the percent beta power is not significantly different, the STA‐LFP magnitude is. This suggests that beta power and STA‐LFP can be decoupled.

From this study, we can conclude that spikes are more synchronized to beta oscillation phase in the STN than in other select structures of PD patients. Spikes are synchronized to beta oscillation phase in both the dorsal and ventral STN in both high and low beta recordings. We also found that high and low percent beta power recordings are equally likely to be found in the dorsal and ventral STN, despite dorsal STN having higher percent beta power both in existing literature and in this study. These findings suggest that spike‐LFP relationship in the STN may be used as another parameter to improve our understanding and treatment of PD movement disorders in the future. Understanding the electrophysiological communication between multiple nuclei in neural circuits can hopefully eventually be expanded to treat other movement and non‐movement disorders.

This study is limited in that it is a correlative study, and it is impossible to determine with certainty whether correlations between single units and LFP oscillations are causative (Teleńczuk et al., 2017). This study also only used one measure, the spike‐triggered average LFP, which can correlate the continuous LFP process with single‐unit activity. This method provides a visual understanding of the correlation, and the differences in magnitude and significant time points can be studied (Snyder & Smith, 2015), but other methods such as spike‐field coherence can also provide a mathematical representation of the degree of correlation (Halliday et al., 1995; Rosenberg et al., 1989).

## 5 FUNDING INFORMATION

The author declares to have received no financial support.

## 6 ETHICS STATEMENT

This study was performed in accordance with the Colorado Multiple Institutional Review Board (COMIRB #16‐1060) and written consent was obtained from all subjects preoperatively.

## Supporting information


Data S1
