# Supplementary material for: Subthalamic nucleus synchronization between beta band local field potential and single‐unit activity in Parkinson's disease
Source: Physiol Rep. 2024 May 2;12(9):e16001. doi: 10.14814/phy2.16001 (PMC11065686; doi:10.14814/phy2.16001)

Subject Demographics

| Subject | Age at time of PD diagnosis | Sex | Years since diagnosis at time of surgery | UPDRS ON/OFF |
| --- | --- | --- | --- | --- |
| 1 | 60 | F | 7 | 13/43 |
| 2 | 64 | M | 4 | 23/41 |
| 3 | 56 | M | 12 | 24/37 |
| 4 | 69 | M | 9 | 22/36 |
| 5 | 53 | M | 14 | 17/42 |
| 6 | 37 | F | 24 | -- |
| 7 | 54 | M | 13 | 20/47 |
| 8 | 61 | M | 5 | 12/23 |
| 9 | 61 | F | 2 | 24/62 |
| 10 | 64 | M | 11 | -- |
| 11 | 70 | F | 5 | 9/29 |
| 12 | 54 | F | 10 | 11/22 |
| 13 | 48 | F | 15 | 12/71 |
| 14 | 71 | M | 6 | 34/49 |
| 15 | 51 | M | 5 | 29/48 |
| 16 | 53 | M | 10 | 29/66 |
| 17 | 60 | M | 12 | 28/35 |
| 18 | 47 | M | 10 | 13/59 |
| 19 | 60 | M | 12 | 28/35 |
| 20 | 55 | M | 12 | 13/26 |
| 21 | 57 | M | 8 | 21/38 |
| 22 | 56 | M | 13 | 24/33 |
| 23 | 55 | F | 7 | -- |
| 24 | 65 | M | 10 | -- |
| 25 | 65 | M | -- | -- |

*UPDRS could not be reliably obtained for some subjects.

Supplementary Figures

S1) Raw local field potential (LFP) tracing showing noise, excluded from analysis by the 300 microvolt exclusion criteria. Background LFP (0-4 seconds and 6-10 seconds) can be distinguished from noise based on appearance and voltage.
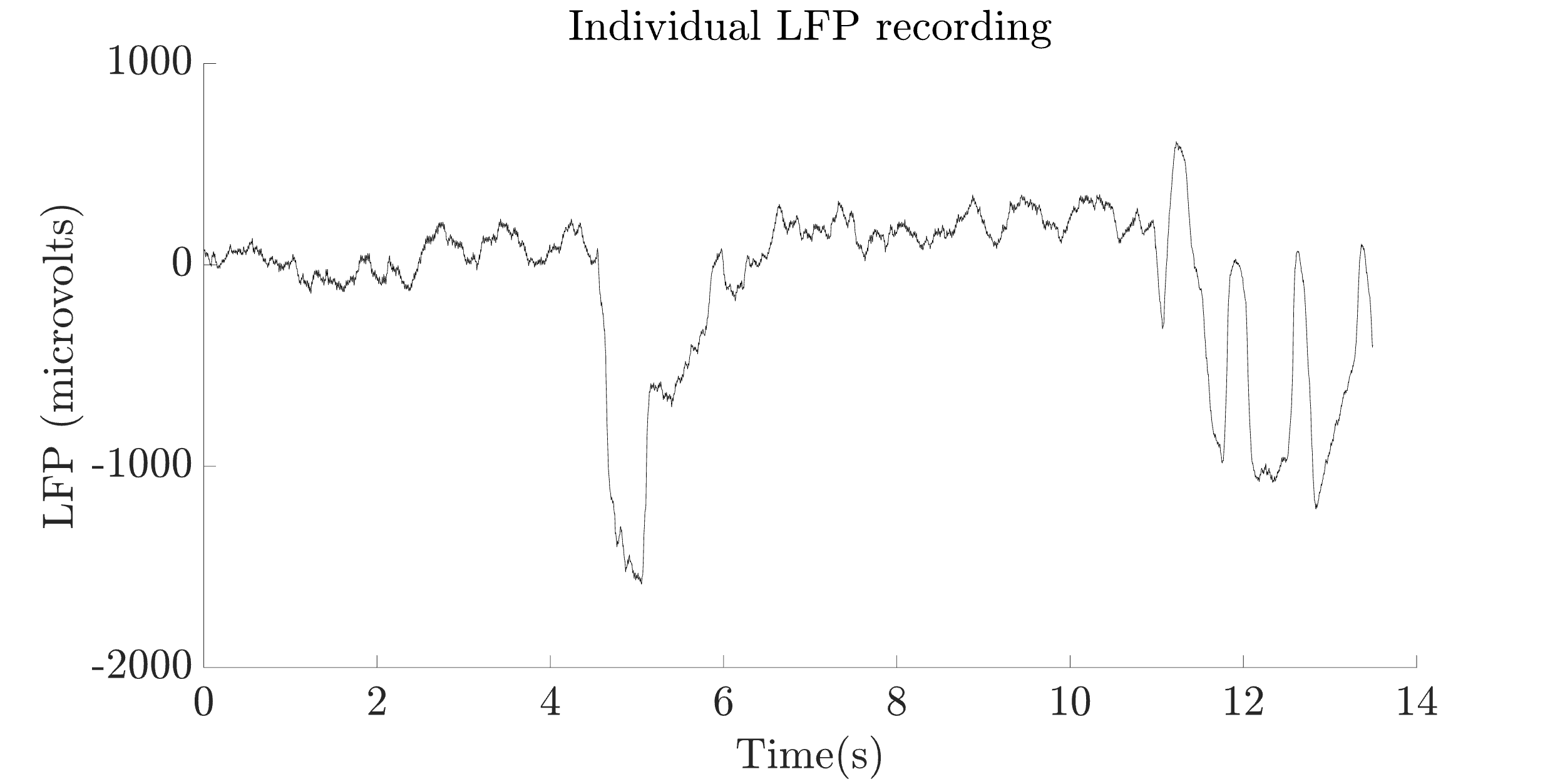


Raw local field potential (LFP) recording contaminated by interference (15-17 seconds), excluded from analysis by the 300 microvolt exclusion criteria.
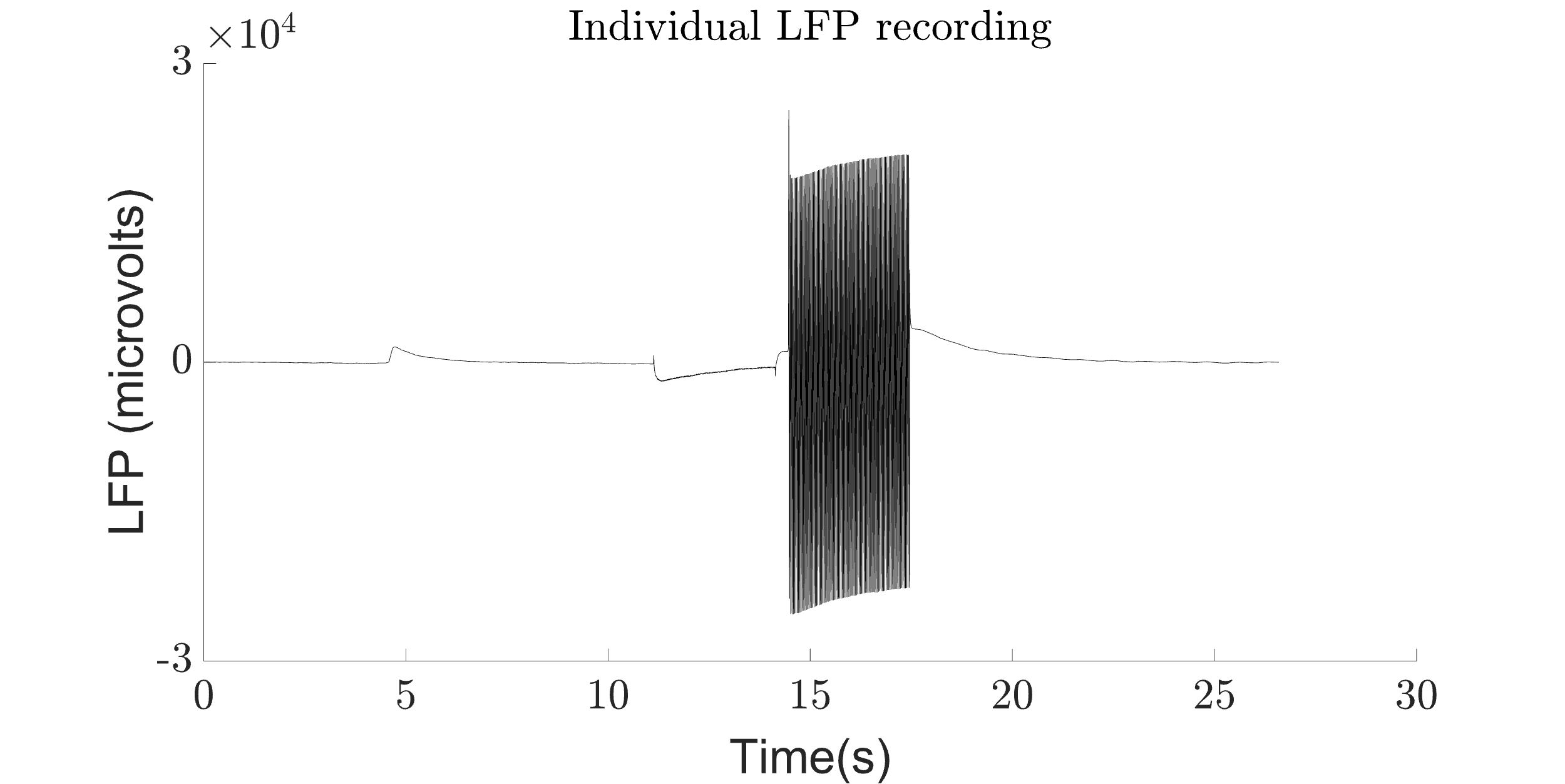


Raw local field potential (LFP) recording contaminated by oscillatory noise at the end of recording. Excluded by the 300 microvolt exclusion criteria.


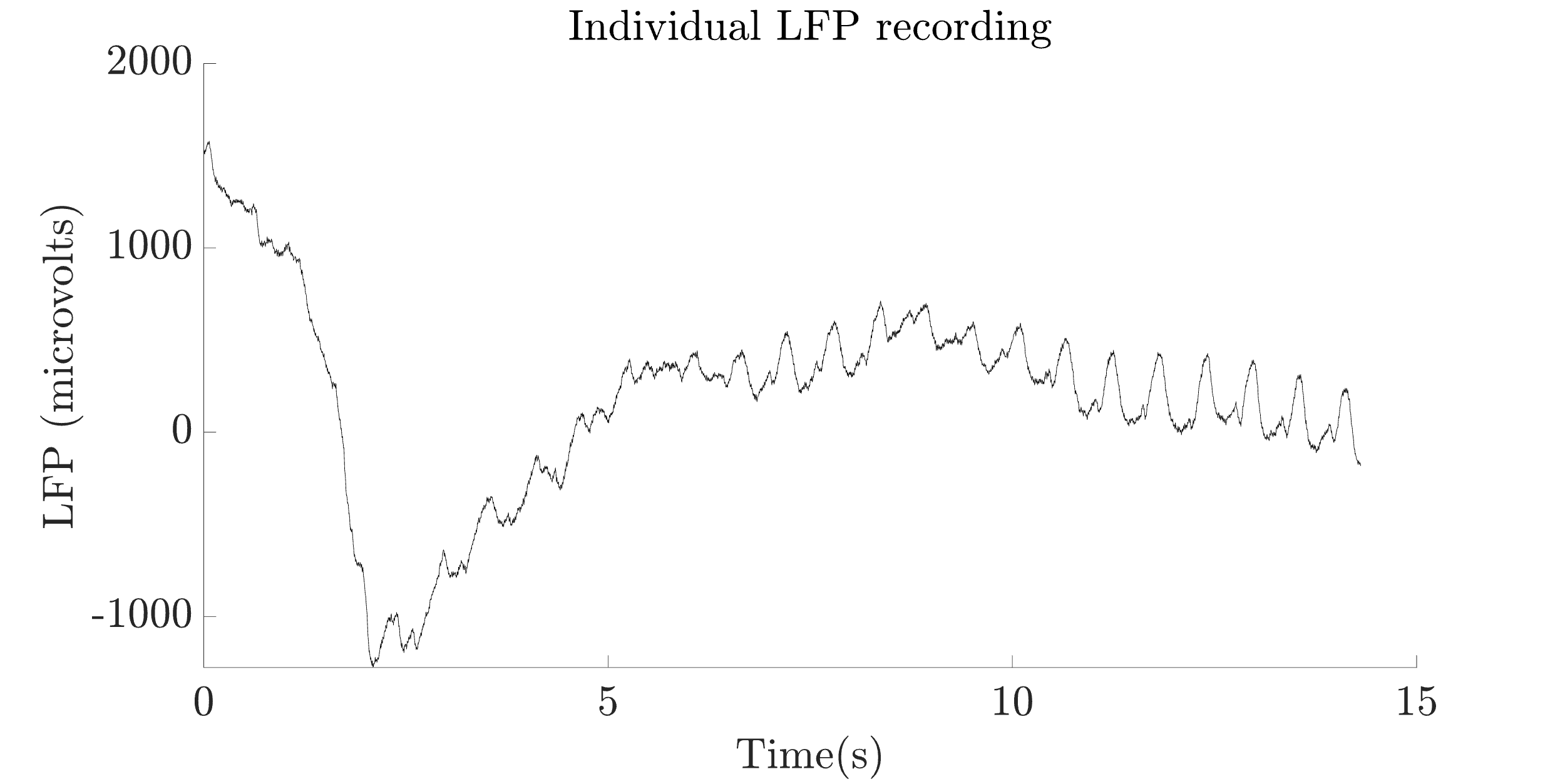


S2) Example of single units sorted using wave_clus. Cluster 1 (blue) was excluded based on waveform and similarity to background noise, confirmed by two evaluators.
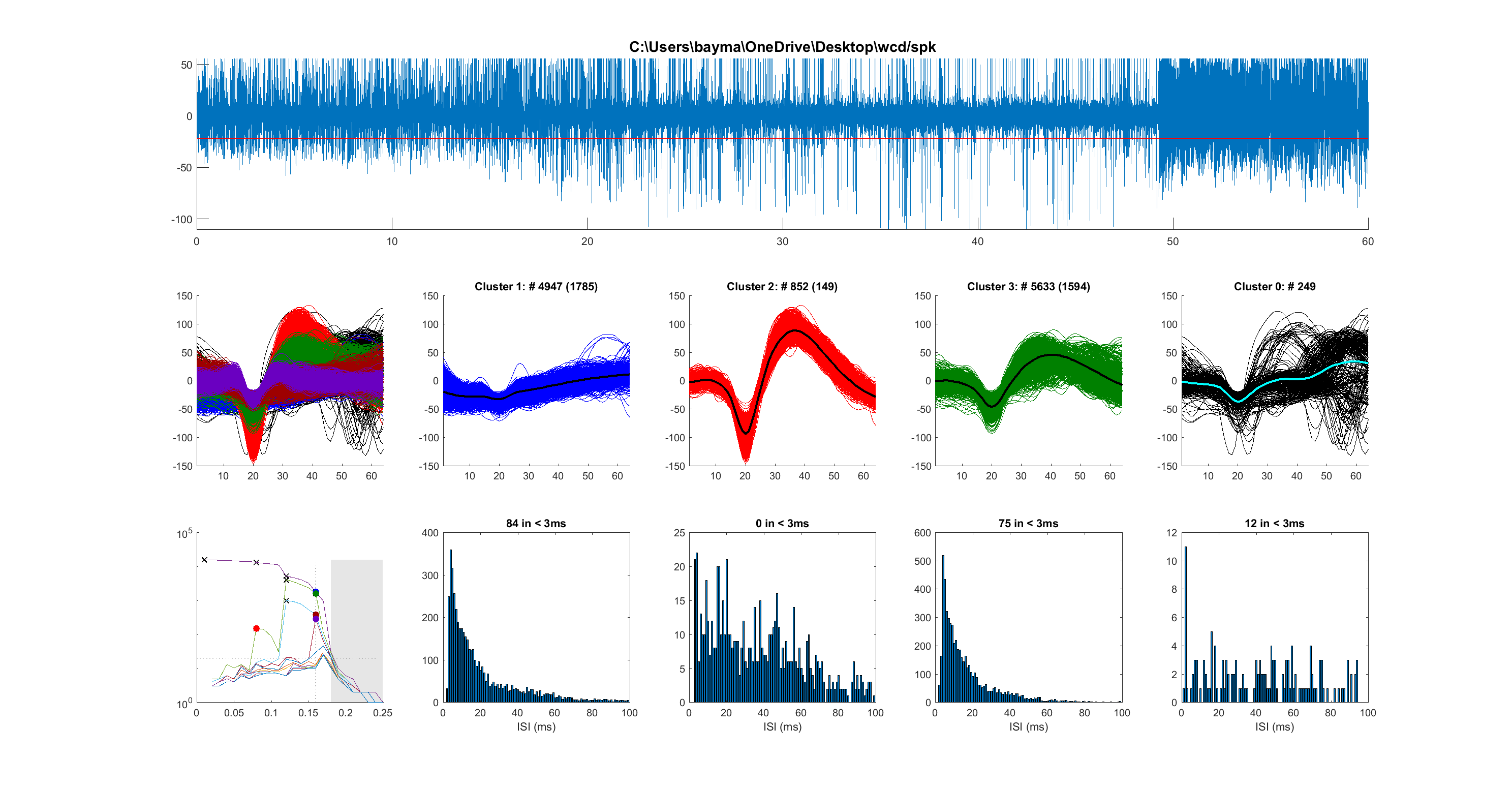


S3) Spike triggered average LFP (STA-LFP), STN vs. other structures, all subjects. Filtered to delta (A), theta (B), alpha (C), beta (D), gamma (E), 0-100Hz (F) frequency bands. Shading represents ± 1 std. error. Legend in F corresponds to A-F. Underlining represents statistically significant time points as in figure 1. Orange & blue: Wilcoxon rank sum tests for high and low percent beta conditions as compared to an STA constructed from randomly chosen spike time points (not pictured as it was near 0 for all conditions tested). Green: Wilcoxon rank sum test between conditions. The largest difference in STA-LFP amplitude (STN vs. Other Structures) can be seen in the beta frequency band

S4) Example of artifact rejection method used to decrease spike contamination of a local field potential recording. Spike is centered at 0ms.
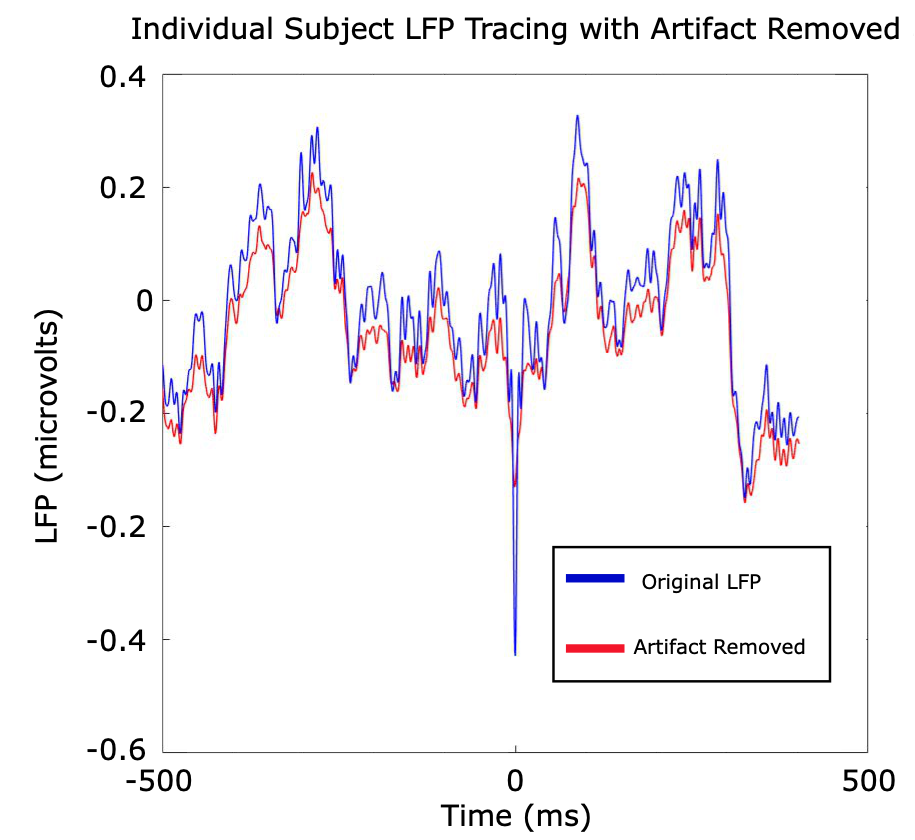

Supplement: Supplementary file 1 — Data S1 [file PHY2-12-e16001-s001.docx]
